# EPSP of *L. casei* BL23 Protected against the Infection Caused by *Aeromonas veronii* via Enhancement of Immune Response in Zebrafish

**DOI:** 10.3389/fmicb.2017.02406

**Published:** 2017-12-08

**Authors:** Chubin Qin, Zhen Zhang, Yibing Wang, Shuning Li, Chao Ran, Jun Hu, Yadong Xie, Weifen Li, Zhigang Zhou

**Affiliations:** ^1^Key Laboratory of Molecular Animal Nutrition, Ministry of Education, College of Animal Science, Zhejiang University, Hangzhou, China; ^2^Key Laboratory for Feed Biotechnology of the Ministry of Agriculture, Feed Research Institute, Chinese Academy of Agricultural Sciences, Beijing, China

**Keywords:** probiotic, *L. casei* BL23, EPSP, *Aeromonas veronii*, immunomodulation

## Abstract

Aquaculture is the fastest-growing food production sector in the world, and it supplies nearly 50% of the global food fish supply. However, disease outbreaks have become a major problem in the fish farming industry. The beneficial contribution of probiotic bacteria to aquatic animals’ health has been widely described, and they have been widely used in aquaculture for disease control and growth promotion. However, the action of probiotic bacterial components and mechanisms underlying protection against pathogens afforded by probiotic bacteria remain poorly understood. In the present study, we pre-colonized zebrafish larvae (before hatching) with 17 potential probiotic bacterial strains and screened for those possessing anti-infective effects against *Aeromonas veronii*. We found that *Lactobacillus casei* BL23 significantly increased the survival of zebrafish larvae upon *A. veronii* infection. Using a germ-free (GF) zebrafish model and gut microbiota transplant experiment, we showed that *L. casei* BL23 *per se* has anti-infective effects in zebrafish larvae, which does not involve microbiota. Furthermore, we identified an exopolysaccharide-protein complex (EPSP) extracted from *L. casei* BL23 cells, which consisted of a 40–45 KD size protein and an exopolysaccharide composed of α-Rha, α-Glc, β-GlcNAc, and β-GalNAc. EPSP significantly increased the survival rate of GF zebrafish at a dose of 10–20 μg/ml after *A. veronii* infection (*P* < 0.01). In addition, the EPSP induced a higher expression of TLR1 and TLR2, and modulated the expression profile of pro-inflammatory and anti-inflammatory cytokines in zebrafish liver (ZFL) cells. Our data indicated that the anti-infective effect of EPSP from *L. casei* BL23 was mediated by enhancement of immune responses in zebrafish, which might involve the TLR1/TLR2 signal pathway.

## Introduction

Aquaculture is the fastest-growing food production sector in the world and a major contributor to global food production, contributing nearly 50% of the global food fish supply (Subasinghe et al., 2009). Aquaculture production of aquatic animals amounted to 73.8 million tons in 2014, with an estimated value of US $160.2 billion. It supplies 17% of animal protein in people’s diets worldwide and supports the livelihoods of about 12% of the world’s population (FAO, 2016). However, disease outbreaks have become a major problem in the fish-farming industry due to the increasing intensification and commercialization of aquaculture practices (Bondad-Reantaso et al., 2005). Infectious diseases have been estimated to cost billions of dollars in the global aquaculture industry annually (Lafferty et al., 2015). For example, the outbreaks of motile *Aeromonas* septicemia (MAS) caused by *Aeromonas* spp. often have high mortality and cause severe economic losses in aquaculture worldwide (Cipriano et al., 1984; Beaz-Hidalgo and Figueras, 2013). During the past few decades, antibiotics have been the standard strategy for management of fish diseases and for improving their growth (Romero et al., 2012). Unfortunately, antibiotic resistance among bacterial pathogens and antibiotic residues in the animal products has piqued global interest in limiting the use of antibiotics in aquaculture (Chen et al., 2015; Huang et al., 2015; Pereira et al., 2015).

Probiotics are live microorganisms that have beneficial effects on the host when properly administered. Extensive studies have demonstrated that probiotics are a promising alternative to antibiotics in aquaculture, and that they have a variety of beneficial effects, including counteraction of dysbiosis, promotion of gut health and homeostasis, promotion of growth, enhancement of immune defenses, and protection of the host from pathogen infection (Newaj-Fyzul et al., 2014; Hai, 2015). Probiotics, especially *Lactobacillus*, have been widely used in aquaculture for disease control, notably against bacterial diseases (Newaj-Fyzul et al., 2014; Fečkaninová et al., 2017). In recent years, there has been increasing interest in determining the biological roles of each probiotic bacterial component. Several factors, i.e., metabolites, enzymes, surface or secreted proteins and cell surface polysaccharides, that influence the immune response of the host have been identified in lactobacilli (Kim et al., 2006, 2009). In particular, studies have indicated that the health benefits of lactic acid bacteria are associated with the production of exopolysaccharides (EPS), which showed antitumor, antiulcer, immunomodulating, and cholesterol-lowering activities (Ruasmadiedo et al., 2002; Welman and Maddox, 2003).

Zebrafish have become a popular model for studying host-bacteria interactions and bacterial pathogenicity (Sullivan and Kim, 2008; Allen and Neely, 2010; Kanther and Rawls, 2010). Zebrafish have an innate immune system and develop adaptive immunity by the age of 4 weeks (Trede et al., 2004; Kanther and Rawls, 2010). In addition, the availability of germ-free (GF) zebrafish larvae combined with available genetic tools make zebrafish particularly suitable for molecular analyses from both the host and bacterial perspectives (Phelps and Neely, 2005; Pham et al., 2008).

In this study, we developed a new experimental approach to direct analysis of bacterial factors involved in the protection of zebrafish larvae by exogenous probiotic bacteria against pathogens. We found *L. casei* BL23 able to robustly protect zebrafish larvae from *A. veronii* infection from 17 potential probiotic bacterial strains. Further, our data indicated that *L. casei* BL23 can enhance host immune responses that may involve the activity of EPSP from BL23 via TLR1/TLR2 pathways.

## Materials and Methods

### Bacteria and Culture Condition

The probiotic strains are listed in **Table 1**. The bacteria were stationarily cultivated in MRS medium at 37°C for 24 h. After growing in MRS medium for 24 h, lactobacilli cells were collected by centrifugation (10 min, 4000 *× g*, 4°C). The pellet was washed by sterile water three times, and resuspended in sterile water at a final concentration of 1.0 × 10^9^ CFU/ml. *A. veronii* was grown in Luria–Bertani (LB) broth for 18 h at 37°C with 200 rpm shaking.

**Table 1:** List of probiotic strains.

Probiotic strains
*Lactobacillus acidophilus* LABCC IMAUFB058
*Lactobacillus casei* LABCC IMAU10005
*Lactobacillus casei* LABCC IMAU10007
*Lactobacillus casei* LABCC IMAU10316
*Lactobacillus casei* LABCC IMAU10325
*Lactobacillus casei* LABCC IMAU10333
*Lactobacillus casei* LABCC IMAU10408
*Lactobacillus casei* BL23
*Lactobacillus rhamnosus* 20300
*Lactobacillus rhamnosus* LGG
*Lactobacillus amylovorus* JCM 1126
*Lactobacillus johnsonii* 466
*Lactobacillus brevis* CGMCC 1.2028
*Lactobacillus plantarum* LABCC IMAU10012
*Lactobacillus plantarum* LABCC IMAU10058
*Lactobacillus plantarum* LABCC IMAU10707
*Lactobacillus plantarum* LABCC IMAU10722

### Animals

Adult zebrafish and larvae (*Danio rerio*) (TU line) were reared in the lab. The adult animals were kept in tanks (length × width × height; 25.5 cm × 18.5 cm × 18.0 cm) in a recirculating aquaculture system under controlled conditions (28 ± 0.5°C, under a 14-h light, 10-h dark photoperiod). The inlet water flow was approximately 1 L/min. The fish were fed twice per day with freshly hatched brine shrimp (8:30 a.m. and 5:30 p.m.). Procedures involving animals were performed in accordance with Chinese legislation associated with animal experimentation and the studies were approved by the Ethics Committee of the Feed Institute, Chinese Academy of Agricultural Sciences (2016-ZZG-ZF-001).

### Probiotics Screening

Probiotic strains were grown stationarily in MRS medium at 37°C for 24 h. Bacteria were then pelleted and washed twice in sterile water, and resuspended in water at a final concentration of 1.0 × 10^7^ CFU/ml. At 3 days post fertilization (dpf), about 12 h before hatching, zebrafish eggs were put in contact with the probiotic strains by transferring them to probiotic-containing bottles (60 eggs per bottle). At 4 dpf, the water was exchanged, and fresh probiotic bacteria cells were added. At 7 dpf, fish were infected with virulent *A. veronii* at a dose of 2 × 10^7^ CFU/ml after water renewal. The mortality was recorded for 5 days.

### GF Zebrafish Husbandry and Gut Microbiota Transplantation

The protocol to generate and rear GF zebrafish was descripted by Pham et al. (2008) with slight modifications. Freshly fertilized zebrafish eggs were washed by sterilized water three times in a 90 mm sterilized dish, and then the eggs were separated into 50 ml Falcon tubes (100 eggs per tube). Eggs were treated with AB-GZM (gnotobiotic zebrafish medium with antibiotics, which contained 250 ng ml^-1^ of amphotericin B, 5 μg ml^-1^ of kanamycin, 100 μg ml^-1^ of ampicillin, and 10 U⋅ mL^-1^ of penicillin and streptomycin) for 4.5 h at room temperature. Then the eggs were washed three times with AB-GZM, and treated with 0.05% of PVPI (polyvinyl pyrrolidone-iodine complex) for 35 s and washed three times with GZM. Next, they were bleached (0.002%) for 15 min. Eggs were washed again three times in GZM and transferred to Petri dishes to be distributed into 300 ml culture bottles with vented caps containing 150 mL of GZM (60 eggs/bottle). GF animals were monitored for sterility every day by spotting 100 μL from each flask on tryptic soy medium agar plates at 28°C under aerobic or anaerobic conditions. Before the gut microbiota transplantation, adult zebrafish were reared in the control or *L. casei* BL23-added water (1.0 × 10^6^ CFU/ml) for two weeks. Then the fish of the control or *L. casei* BL23 treatment group were sacrificed and the intestinal contents of every five fish from each group were pooled. Three replicate bottles of GF zebrafish larvae (4 dpf, *n* = 60) were transplanted with gut microbiota of control or BL23-treated fish at a dose of 10^5^ and 10^6^ CFU/mL. After 3 days of colonization, fish were challenged with *A. veronii* at a dose of 2.0 × 10^7^ CFU/mL after GZM was renewed. The zebrafish mortality was observed for 120 h after infection.

### DNA Extraction and Sequencing

Total bacteria DNA was extracted from intestinal contents samples by using Power Fecal^TM^ DNA Isolation kit (MO BIO Laboratories, Carlsbad, CA, United States) according to manufacturer’s instruction. Sequencing was performed at the Novogene Bioinformatics Technology Co., Ltd. Briefly, DNA was amplified by using the 515F/806R primer set (341F: 5′-CCTAYGGGRBGCASCAG-3′ 806R: 5′-XXXXXXGGACTACHVGGG TWTCTAAT-3′), which targets the V-V43 region of the bacterial 16S rDNA, with the reverse primer containing a 6-bp error-correcting barcode unique to each sample. PCR reaction was performed using phusion high-fidelity PCR Mastermix (New England Biolabs LTD., Beijing, China) with the following condition: 94°C for 5 min (1 cycle), 94°C for 20 s/55°C for 20 s/72°C for 30 s (30 cycles), and a last step of 72°C for 10 min. PCR products were purified by using the QIAquick Gel Extraction Kit (QIAGEN, Dusseldorf, Germany). Pyrosequencing was conducted on an Illumina HiSeq 2500 platform according to protocols described by Caporaso et al. (2012). Paired-end reads were merged using FLASH^1^ (V1.2.7) (Magoc and Salzberg, 2011). Sequences were then demultiplexed and quality filtered using the default parameters of the Quantitative Insights into Microbial Ecology (QIIME) software package (Caporaso et al., 2010). The operational taxonomic unit (OTU) clustering pipeline UPARSE was used to select OTUs at 97% similarity (Edgar, 2013). The representative sequence sets were aligned and given a taxonomic classification using Ribosomal Database Project (Wang et al., 2007). The similarity among microbial communities was determined using histograms, UniFrac principal coordinates analysis (PCoA), and the unweighted pair-group method with arithmetic mean (UPGMA).

### *L. casei* BL23 Exposure

*Lactobacillus casei* BL23 cells were grown stationarily in MRS medium at 37°C for 24 h. Bacteria were then pelleted and washed twice in sterile water, and resuspended in sterile water at a final concentration of 1.0 × 10^9^ CFU/ml. To obtain dead cells, the bacterial pellets were treated with 4% paraformaldehyde for 2 h, and washed three times in sterile water. After hatching (4 dpf), conventional or GF zebrafish were exposed with live or dead cells of *L. casei* BL23 at a dose of 1 × 10^5^ CFU/ml, 1 × 10^6^ CFU/ml and 1 × 10^7^ CFU/ml, respectively for 3 days. After three days of probiotics exposure, fish were then infected with virulent *A. veronii* at a dose of 2 × 10^7^ CFU/ml after GZM was renewed. The mortality was recorded for 5 days. At the infected time of 0, 24, and 48 h, thirty fish (GF and fish treated with live cells or dead cells of *L. casei* BL23 at the dose of 1 × 10^6^ CFU/ml) from each culture bottle were sacrificed and the whole body was sampled. The samples were immediately frozen in liquid nitrogen and stored at -70°C for cytokine expression analysis.

### EPSP Preparation and Characterization

The method to extract EPSP was previously described by Zhang et al. (2016) in our lab. *L. casei* BL23 cells were grown stationarily in MRS medium at 37°C for 48 h. Bacteria were then pelleted and washed twice in sterile water, and were resuspended in sterile water. Then bacteria were incubated in water bath incubation (70°C, 24 h). The extracts were precipitated by gradually adding cold ethanol to 75% (v/v), and the supernatant was removed after 24 h, followed by centrifugation at 12000 rpm for 20 min. The precipitated product was washed and dissolved in water obtained from an Alpha-Q reagent grade water purification system (Millipore Co., Milford, MA, United States). The aqueous solution of the extracts were further treated with sevage reagent (trichloromethane – *n*-butanol, 4:1, vol/vol) at a final concentration of 25% and incubated for 2 h under gentle agitation and then precipitated proteins were removed by centrifugation at 8000 *g* for 20 min (repeat this step for two times). After centrifugation, the solution containing EPS was dialyzed (molecular weight cut-off: 3000 Da) against 5 l of distilled water for 2 days with water changes three times per day. The extract solution after dialysis was lyophilized.

The purity of the extract (5 mg/ml) was tested by SDS-PAGE electrophoresis and size-exclusion chromatography (SEC) on a column of Superdex75 (10/300 GE) (Pharmacia, Uppsala, Sweden), which fitted to an AKTA FPLC system (Pharmacia) and were eluted with 0.3 M NaCl buffer.

The monosaccharide composition was determined by TLC. Briefly, 20 mg EPSP was hydrolyzed with 2 ml sulfuric acid (1 mol/l) at 100°C for 4 h. The residual sulfuric acid was removed by neutralization with excessive BaCO_3_ reaction for 12 h. This solution was adjusted to pH7 and diluted to 20 ml. The hydrolyzate was evaporated under reduced pressure, dissolved in 2 ml ultra-pure water. The resulting hydrolyzate was analyzed by TLC. TLC analysis was conducted according to the previous report (Tanaka et al., 1999). Migration was performed twice on a silica gel TLC plate (20 cm × 20 cm) using *n*-butanol–methanol–25% ammonia solution–water (5:4:2:1 [vol/vol/vol/vol]). Carbohydrates were visualized by heating the TLC plate after spraying with aniline-diphenylamine reagent (4 ml of aniline, 4 g of diphenylamine, 200 ml of acetone, and 30 ml of 85% phosphoric acid). Monosaccharides of α-Rha, α-Glc, β-GlcNAc, and β-GalNAc were used as standard and the plate was baked at 110°C for 5 min.

### Cell Culture and Treatments

The cell line of ZFL was purchased from American Type Culture Collection (ATCC). The ZFL cells were cultured at 28°C in modified limit dilution factor (LDF) culture medium. The complete medium consisted of 50% Leibovitz’s L-15 (L-15), 30 % Dulbecco’s modified Eagle’s (DMEM), and 20% Dulbecco’s Modification of Eagle’s Medium/Ham’s F-12 medium (DMEM/F12) supplemented with 5% fetal bovine serum (FBS), 0.5% trout serum, 10 μg/ml bovine insulin, 50 ng/ml mouse Epidermal Growth Factor (EGF). Mediums were supplemented with 1% penicillin–streptomycin. All basal mediums and FBS were obtained from Corning (NY, United States). The cells were treated with EPSP (10 μg/ml) or equal volume of dd H_2_O after the cells covered the plate, and cells were harvested at 24 h after treatment.

### Real-Time PCR

Total RNA was isolated from zebrafish larvae and ZFL cells with TRIzol (Invitrogen) extraction. First-strand complementary DNA synthesis was performed using the Superscript First-Strand Synthesis System (Invitrogen). Quantitative real-time PCR reaction were performed using the Power SYBR Green PCR Master Mix (Applied Biosystems) on an ABI 7500 (Applied Biosystems) with reaction volumes of 20 μl. The reaction mixtures were incubated for 5 min at 95°C, followed by 40 cycles of 20 s at 95°C, 20 s at 60°C and 20 s at 72°C, and finally the melt curve was performed from 65to 95°C with a 0.5°C increment for 10 s. Two genes, including rpl13 and rps11 were used as references. The primer sequences are listed in **Table 2**.

**Table 2:** Primers for RT-Qpcr.

Primer	Sequence (5′–3′)
rps11 F	ACAGAAATGCCCCTTCACTG
rps11 R	GCCTCTTCTCAAAACGGTTG
rpl13 F	TCTGGAGGACTGTAAGAGGTATGC
rpl13 R	TCAGACGCACAATCTTGAGAGCAG
TNF-α F	CAGAGTTGTATCCACCTGTTA
TNF-α R	TTCACGCTCCATAAGACCCA
IL-10 F	ATTTGTGGAGGGCTTTCCTT
IL-10 R	AGAGCTGTTGGCAGAATGGT
Saa F	CGCAGAGGCAATTCAGAT
Saa R	CAGGCCTTTAAGTCTGTATTTGTTG
IL-1β F	GAGACAGACGGTGCTGTTTA
IL-1β R	GTAAGACGGCACTGAATCCA
TLR-4a F	TGTCAAGATGCCACATCAGA
TLR-4a R	TCCACAAGAACAAGCCTTTG
TLR3 F	CTACGTGATAGCTCCGCCTC
TLR3 R	ACAAGCGTAGAACAAGGGCA
TLR5a F	CATTCTGGTGGTGCTTGTT
TLR5a R	CTGCTGCTTCAGGATTGTT
TLR2 F	ATACAAGCCAAACGGAAACCT
TLR2 R	CTTCTCACATTTCCGCATCAT
NF-κB F	GCAAGATGAGAACGGAGACAC
NF-kB R	CTACCAGCAATCGCAAACAA
TLR5b F	GTGAGGAGCCTGATCCTGATAG
TLR5b R	CATACTAAATGTATAATAAGTCTACCATG
Myd88 F	TCCACAGGGACTGACACCTGAGA
Myd88 R	GCTGAGTCTTCAGCACAGCAGAT
TLR1 F	CCCAAGCTTGAAGGCGACTGTG
TLR1 R	GTACTTTGAGGGAATGAGATACAG
IL-6 F	TCAACTTCTCCAGCGTGATG
IL-6 R	TCTTTCCCTCTTTTCCTCCTG

### Statistical Analysis

Animal survival rates were analyzed by Kaplan–Meier survival estimate with Bonferroni *post hoc* test with GraphPad Prism version 5.0 software. Other data were analyzed by one-way analysis of variance (ANOVA) followed by Bonferroni *post hoc* test with GraphPad Prism version 5.0 software. In addition, unpaired *t*-test was used to compare data from two groups when appropriate. Wherever applicable, *P*-values are reported, and a *P*-value of ≤0.05 is considered significant.

## Results

### Identification of Probiotic Bacteria That Mitigate *A. veronii* Infection in Pre-colonized Zebrafish Larvae

In order to screen the probiotic strains that protect zebrafish larva from damage induced by *A. veronii* infection, we pre-colonized unhatched (3 dpf) conventional zebrafish larvae with 17 Gram-positive bacteria commonly often used as probiotics in aquaculture and elsewhere in the food industry (**Table 1**). These pre-colonized larvae were then infected at 7 dpf with *A. veronii* and their mortality rate was compared to that of the control larvae. The result showed that pre-incubation with *Lactobacillus casei* BL23 significantly increased the survival rate of larvae upon *A. veronii* infection (**Supplementary Figure S1**).

### Assessment of *L. casei* BL23 Protection against *A. veronii* Infection in Zebrafish Larvae by Dose

**FIGURE 1:**
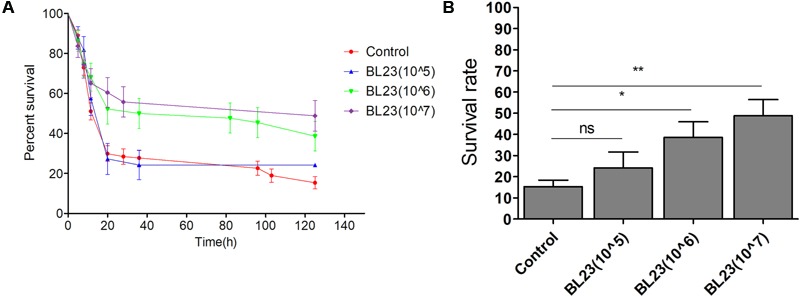
Dose dependent of *Lactobacillus casei* BL23 protection against *Aeromonas veronii* infection in conventional zebrafish larvae. The survival rate **(A)** and final survival rate **(B)** of zebrafish larvae treated with *L. casei* BL23 at a dose of 10^5^, 10^6^, and 10^7^ CFU/ml, respectively, after infected with *A. veronii*. Asterisks indicate significant difference compared with control (^∗^*P* < 0.05, ^∗∗^*P* < 0.01, ^∗∗∗^*P* < 0.001).

To characterize the protective effect of *L. casei* BL23 in zebrafish larvae, we first determined whether the protective effect towards zebrafish larvae was dose-dependent. As shown in **Figures 1A,B**, zebrafish larvae pre-colonized with increasing dosage of *L. casei* BL23 correlated with increased larvae survival rate after *A. veronii* infection. No significant difference in survival rate was observed between the larvae pre-colonized with *L. casei* BL23 at the dose of 1.0 × 10^5^ CFU/ml and control larvae after *A. veronii* infection (**Figures 1A,B**). However, the survival rate of larvae pre-colonized with BL23 at 1.0 × 10^6^ CFU/ml or 1.0 × 10^7^ CFU/ml was significantly higher than that of control larvae after *A. veronii* infection (**Figures 1A,B**, *P* < 0.05). These data indicated that the protective effect of *L. casei* BL23 in zebrafish was dose dependent.

### The Protection of *L. casei* BL23 in Zebrafish Was Mediated by the Bacteria Itself and Did Not Involve the Microbiota

**FIGURE 2:**
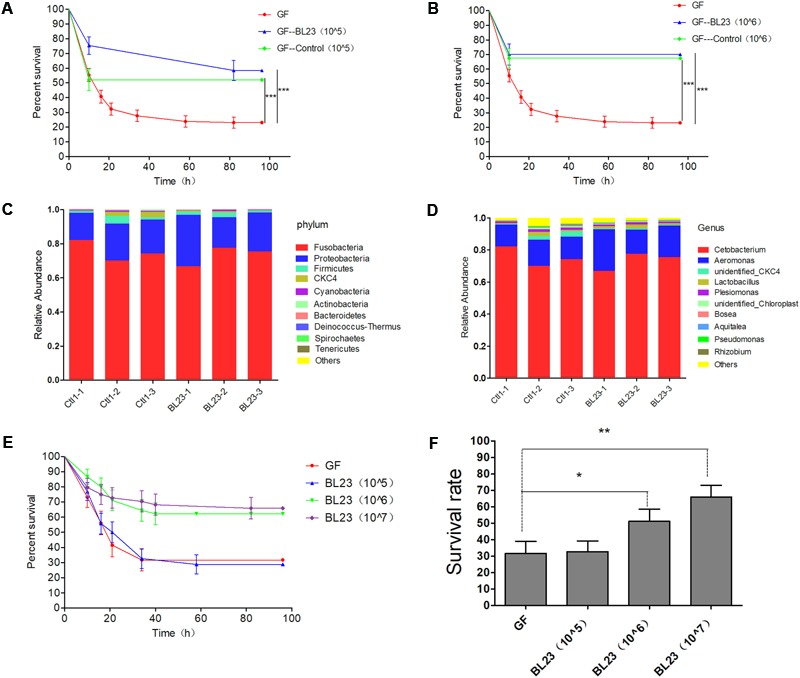
The protection of *L. casei* BL23 was mediated by the bacteria itself and does not involve intestinal microbiota of host. The survival rate of GF zebrafish larvae colonized with gut microbiota from control or *L. casei* BL23 treated fish at a dose of 10^5^ CFU/mL **(A)** and 10^6^ CFU/mL **(B)** after infected with *A. veronii*. Gut microbiota of zebrafish from control or *L. casei* BL23 treated fish at phylum **(C)** and genus **(D)** level. The survival rate **(E)** and final survival rate **(F)** of GF zebrafish larvae treated with *L. casei* BL23 at a dose of 10^5^, 10^6^, and 10^7^ CFU/Ml, respectively after infected with *A. veronii*. Asterisks indicate significant difference compared with control (^∗^*P* < 0.05, ^∗∗^*P* < 0.01, ^∗∗∗^*P* < 0.001).

In order to determine whether the protective effect of *L. casei* BL23 on zebrafish is affected by the bacterium itself or via alterations in the gut microbiota, a GF zebrafish and gut microbe transplant model was established. Here, the intestinal microbiota associated with administration of control treatment and *L. casei* BL23 treated (*L. casei* BL23 administration for 2 weeks at a dose of 1.0 × 10^6^ CFU/ml) zebrafish were transferred to freshly hatched GF zebrafish at 4 dpf. These gut microbiota recipient larvae were then infected with *A. veronii* at 7 dpf and their mortality was compared to that of the *A. veronii*-infected GF larvae. The results showed survival to be significantly higher in zebrafish colonized with microbiota from either the control or fish treated with *L. casei* BL23 at doses of 1.0 × 10^5^ CFU/ml and 1.0 × 10^6^ CFU/ml compared with the GF fish (**Figures 2A,B**, *P* < 0.001). No significant difference in survival rate was observed between larvae colonized with microbiota from control or BL23-treated zebrafish (**Figures 2A,B**).

High-throughput sequencing with the 16S *r*RNA gene was performed to characterize the gut microbiota of zebrafish treated with the control or *L. casei* BL23. The results showed that phyla Fusobacteria and Proteobacteria and genus of *Cetobacterium* and *Aeromonas* were dominant in the intestines of zebrafish (**Figures 2C,D**). The gut microbial community exhibited no statistical difference between the two groups at the level of phylum to genus (**Figures 2C,D** and **Supplementary Figures S2A–C**). These data indicated that administration of *L. casei* BL23 did not alter the gut microbiota in zebrafish, which is consistent with the results of the microbiota transfer experiment described above.

**FIGURE 3:**
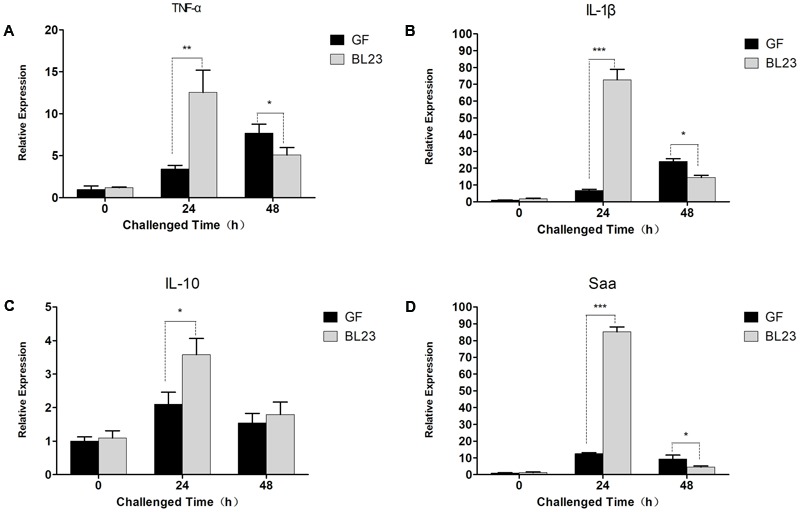
*Lactobacillus casei* BL23 modulated immune response against *A. veronii* infection in zebrafish. The mRNA levels of TNF-α **(A)**, IL-1β **(B)**, IL-10 **(C)**, and Saa **(D)** from GF and *L. casei* BL23(10^6^ CFU/mL) treated zebrafish at certain time points after infected with *A. veronii*. The results are given as mean ± SEM (*n* = 3). Asterisks indicate significant difference compared with control (^∗^*P* < 0.05, ^∗∗^*P* < 0.01, ^∗∗∗^*P* < 0.001).

We then mono-colonized GF zebrafish larvae (4 dpf) with *L. casei* BL23, then the larvae were infected with *A. veronii* at 7 dpf. We found that the survival of BL23-treated GF larvae was significantly higher compared with the GF larvae after challenge with *A. veronii*. The protection mediated by BL23 was found to be dose-dependent (**Figures 2E,F**, *P* < 0.001). Among larvae treated with *L. casei* BL23, the transcription levels of several chemokines, specifically interleukin-1β (IL-1β), tumor necrosis factor α (TNF-α), interleukin-10 (IL-10), and serum amyloid A (Saa), increased 4–80 fold at 24 h after *A. veronii* infection, but it decreased to near basal levels at 48 h (**Figure 3**). In contrast, the transcription level of these cytokines in GF fish increased slowly and the increasing trend was maintained for 48 h post challenge (**Figure 3**). The mRNA levels of TNF-α, IL-1β, IL-10, and Saa were higher in *L. casei* BL23-treated larvae than in GF larvae at 24 h (**Figures 3A–D**, *P* < 0.01) and lower (except IL-10) at 48 h after challenge with *A. veronii* (**Figures 3A,B,D**, *P* < 0.05).

Collectively, these data indicated that the protection of *L. casei* BL23 against *A. veronii* infection in zebrafish larvae was mediated by baceterium itself and did not involve the microbiota.

### Protective Effect of *L. casei* BL23 Irrespective of Cells’ Viability

**FIGURE 4:**
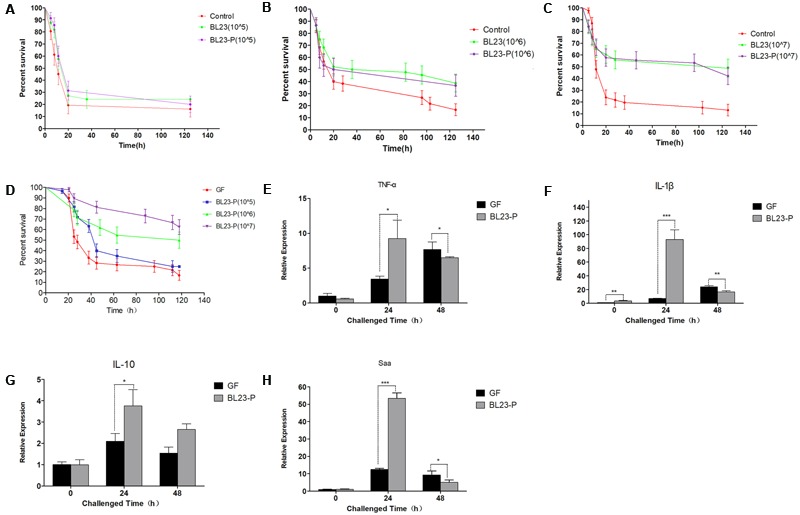
Anti-infective activity of *L. casei* BL23 was mediated by some cellular structural components irrespective of cell viability. The survival rate **(A)** of conventional zebrafish larvae treated with live or dead cells of *L. casei* BL23 at a dose of 10^5^ CFU/ml after infected with *A. veronii*. The survival rate **(B)** of conventional zebrafish larvae treated with live or dead cells of *L. casei* BL23 at a dose of 10^6^ CFU/ml after infected with *A. veronii*. The survival rate **(C)** of conventional zebrafish larvae treated with live or dead cells of *L. casei* BL23 at a dose of 10^7^CFU/ml after infected with *A. veronii*. The survival rate **(D)** of GF zebrafish larvae treated with dead cells of *L. casei* BL23 at a dose of 10^5^, 10^6^, and 10^7^ CFU/Ml, respectively after infected with *A. veronii*. The mRNA levels of TNF-α **(E)**, IL-1β **(F)**, IL-10 **(G)**, and Saa **(H)** from GF and dead BL23 (10^6^ CFU/mL) treated zebrafish at certain time points after infected with *A. veronii*. Asterisks indicate significant difference compared with control (^∗^*P* < 0.05, ^∗∗^*P* < 0.01, ^∗∗∗^*P* < 0.001).

In order to determine whether protective effect of *L. casei* BL23 in zebrafish is mediated by cellular metabolites or cell structural components, we tested the effect of live and dead (4% paraformaldehyde fixed) cells of *L. casei* BL23 in both conventional and GF zebrafish. The results showed that, for both conventional and GF zebrafish, live and dead cells of *L. casei* BL23 both efficiently increased the survival rate of zebrafish larvae after infection with *A. veronii* (**Figures 4B–D**, 2E,F, *P* < 0.05). In addition, a similar difference in cytokine expression was observed between dead BL23-treated larvae and live BL23-treated larvae after *A. veronii* infection (**Figures 4E–H**). These data suggest that the immuno-regulation and anti-infectious activity of *L. casei* BL23 was mediated by certain cell components irrespective of cell viability.

### EPS Extract from *L. casei* BL23 and *A. veronii* Infection

**FIGURE 5:**
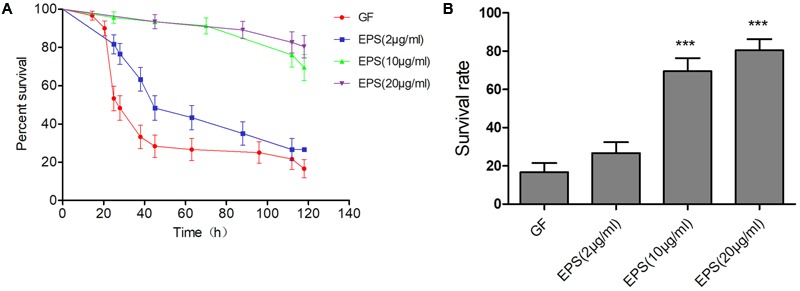
Exopolysaccharides (EPS) extracted from *L. casei* BL23 protected against *A. veronii* infection in GF zebrafish larvae. The survival rate **(A)** and final survival rate **(B)** of GF zebrafish larvae treated with EPSP at a dose of 2, 10, and 20 μg/mL, respectively after infected with *A. veronii*. Asterisks indicate significant difference compared with control (^∗^*P* < 0.05, ^∗∗^*P* < 0.01, ^∗∗∗^*P* < 0.001).

Using the data given above, we speculated that the protective effect of *L. casei* BL23 in zebrafish might involve the EPS of the bacteria. So, we extracted and tested the biological roles of the EPS from *L. casei* BL23. GF larvae (4 dpf) were treated with 2, 10, and 20 μg/ml EPS from *L. casei* BL23, respectively. Then the larvae were infected with *A. veronii* at 7 dpf, and the survival rates of these fish were compared to the *A. veronii*-infected GF fish. The survival rate of fish treated with the *L. casei* BL23 EPS at 10 and 20 μg/ml was significantly higher than those of GF fish after *A. veronii* infection (**Figures 5A,B**, *P* < 0.01).

### Extraction and Characterization of the EPS Extract from *L. casei* BL23

**FIGURE 6:**
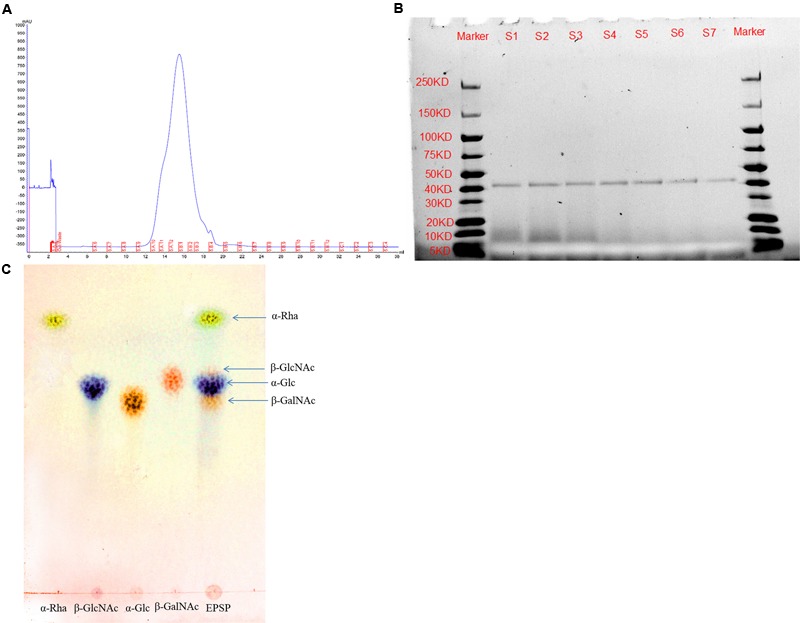
Characterization of the EPSP from *L. casei* BL23. Purity of EPSP analyzed by size-exclusion chromatography (SEC) on a column of Superdex75 (10/300 GE) **(A)** and SDS-PAGE **(B)**. Monosaccharide composition of EPSP (α-Rha, α-Glc, β-GlcNAc, and β-GalNAc) analyzed by TLC **(C)**.

The EPS extract was purified by SEC on a column of Superdex75 (10/300 GE). Interestingly, as shown in **Figure 6A**, the EPS extract showed a single, symmetrical protein peak, indicating that the EPS extract contains a homogeneous protein. It was also confirmed by the SDS-PAGE analysis, which showed a single band with 40–45 KD in size (**Figure 6B**). In addition, qualitative carbohydrate analysis of the fractions (purified by SEC) with the phenol–sulfuric acid method showed that all the positive reactions were within the peak of the protein fractions. Furthermore, the monosaccharide composition of the EPS extract was determined by TLC after acid hydrolysis. TLC indicated that the EPS of the cell extract from *L. casei* BL23 was composed of α-Rha, α-Glc, β-GlcNAc, and β-GalNAc (**Figure 6C**). Together, these findings indicated that the EPS extract is an exopolysaccharide-protein complex (EPSP).

### Immuno-regulation of EPSP in ZFL Cells

**FIGURE 7:**
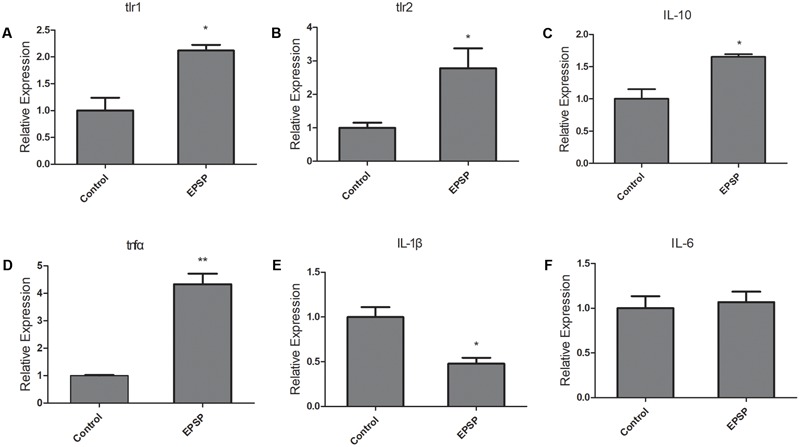
Exopolysaccharide-protein complex (EPSP) modulated immune response in ZFL cells via TLR1 and TLR2. The mRNA levels of TLR1 **(A)**, TLR2 **(B)**, IL-10 **(C)**, TNFα **(D)**, IL-1β **(E)**, and IL-6 **(F)** in ZFL cells from control and EPSP treatment (10μg/mL) groups after 24 h treatment. Asterisks indicate significant difference between the control and EPSP treatment group (^∗^*P* < 0.05, ^∗∗^*P* < 0.01, ^∗∗∗^*P* < 0.001).

In order to characterize EPSP-mediated immuno-regulation in zebrafish, we tested the immune response of ZFL cells after EPSP treatment. The transcription level of toll-like receptors (TLRs) and cytokines were evaluated in ZFL cells at 24 h after EPSP treatment. The results showed that EPSP induced more expressionl of TLR1 and TLR2 compared with the control (**Figures 7A,B**, *P* < 0.05). In addition, the transcription level of IL-10 and TNF-α was significantly higher in ZFL cells treated with EPSP than in the control (**Figures 7C,D**, *P* < 0.05). Gene expression of IL-1β was less pronounced than in the control after EPSP treatment in ZFL cells (**Figure 7E**, *P* < 0.05). No significant difference between the EPSP-treated and control ZFL cells was observed in the transcription levels of TLR3, TLR4a, TLR5a, TLR5b, MyD88, NF-κB, or IL-6 (**Figure 7F** and **Supplementary Figures S3A–G**). These data suggested that the immuno-regulation of EPSP on zebrafish were might involve the TLR1/TLR2 signal pathway.

## Discussion

Infectious diseases remain the major problem in aquaculture production and food security (Leung et al., 2013; Stentiford et al., 2017). Industry-wide losses to aquatic animal diseases exceed 6 billion dollars per year (World Bank, 2014). In certain sectors (e.g., farming of shrimp and various fishes), disease outbreaks have particularly devastating economic and social impacts, with total losses exceeding 40% of the worldwide capacity (Israngkura and Haesae, 2002; Lafferty et al., 2015).

Probiotics have been widely used in aquaculture for disease control and immune improvement for decades (Newaj-Fyzul et al., 2014). However, several studies and meta-analyses of randomized probiotic trials have shown that application of probiotics has varying success rates (Sazawal et al., 2006; Kalliomaki et al., 2010; Li-Li, 2010). This is in part because the efficacy of different probiotic strains against particular pathogens is often both host-specific and probiotic strain-specific. Moreover, knowledge concerning the precise molecular mechanisms underlying the action of specific probiotic strains is limited (Nayak, 2010). In this study, we showed that among 17 selected probiotic strains, only *L. casei* BL23 was able to protect zebrafish from *A. veronii* infection (**Figure 1**).

Possible benefits of probiotic treatment have already been suggested: competitive exclusion of pathogenic bacteria, production of inhibitory compounds, inhibition of the expression of virulence genes, disruption of quorum sensing of the pathogens, improvement in water quality, and enhancement of the immune response against pathogens (Pandiyan et al., 2013; Fuente Mde et al., 2015; Reddy, 2015; Selvaraju, 2015). Here, we developed a GF zebrafish and gut microbiota transplant model to characterize the mode of action of *L. casei* BL23 in protecting zebrafish from *A. veronii* infection. The results showed that the gut microbial community exhibited no statistically significant difference between *L. casei* BL23 administration group and control group at the level of phylum to genus (**Figures 2C,D** and **Supplementary Figures S2A–C**). This might be because *L. casei* BL23 does not colonize the intestine of zebrafish (Qin et al., 2014). In addition, we showed that both live and dead cells of *L. casei* BL23 significantly increased resistance against *A. veronii* infection in GF and conventional zebrafish (**Figures 2E,F, 4**, *P* < 0.05). *L. casei* BL23 also modulated the expression of the pro-inflammatory cytokines IL-1β and TNF-α, the inflammation marker Saa, and the regulator cytokine IL-10 after *A. veronii* infection (**Figure 3**, *P* < 0.05). We speculated that the anti-infective effect of *L. casei* BL23 was mediated by the enhancement of immune responses against pathogens in zebrafish, which was induced by certain cellular structural components of BL23, but not mediated by metabolites. We also speculated that this anti-infective effect of *L. casei* BL23 did not involve alteration of the gut microbiota.

Bacterial cell components such as peptidoglycan (PGN) (Mackenzie, 2010), lipoteichoic acid (LTA) (Grangette et al., 2005), EPS (Vinderola et al., 2006; Wu et al., 2010), outer membrane proteins (OMP), and extracellular proteins (ECP) (Abbass et al., 2010; Sharifuzzaman et al., 2011) from Gram-positive bacteria have been reported to act as potent immunostimulants for animals. These molecules possess conserved microbe-associated molecular patterns (MAMPs), which can be recognized by pattern recognition receptors (PRRs), e.g., TLRs, nucleotide oligomerization domain (NOD)-like receptors (NLRs), and C-type lectin receptors (CLRs). They then modulate the immune response of the host (Lebeer et al., 2010; Bron et al., 2012).

Exopolysaccharides produced by some strains of LAB have been shown to possess beneficial health effects, such as blood cholesterol reduction (Nakajima et al., 2010), immunostimulatory capacities (Chabot et al., 2001a), and antitumor activity (Kitazawa et al., 1998). For example, the antihypertensive and anti-infective activities of strain *L. casei* YIT9018 were attributed to polysaccharide–glycopeptide and polysaccharide–peptidoglycan (PS-PG) complexes, respectively (Nagaoka et al., 1990; Sawada et al., 1990). Immunomodulating properties were also reported for the cell wall polysaccharide (WPS) of *L. casei* strain Shirota (YIT 9029) (Emi et al., 2008). Kefiran, an EPS produced by a number of strains of lactobacilli in the fermented milk drink kefir, may play a role in promoting intestinal homeostasis by increasing luminal IgA and both pro- and anti-inflammatory cytokines, such as IFN-c, TNFα, IL-6, and IL-10, as observed in the small and large intestine (Vinderola et al., 2006). Additionally, EPSs isolated from strains of lactobacilli and bifidobacteria have been found to augment the release of both pro- inflammatory and anti-inflammatory cytokines, such as TNF-α, IL-6, and IL-10 in murine macrophages (Chabot et al., 2001b; Bleau et al., 2010; Wu et al., 2010). Chabot et al. (2001a) suggested EPS could exert their action via the mannose receptor. Lin et al. (2011) reported that TA-1 (novel EPS) can stimulate the release of the pro-inflammatory cytokines TNF-α and IL-6 from murine macrophages via a TLR2 mediated pathway.

In the present study, EPSP extracted from *L. casei* BL23 was characterized and consists of EPS and a 40–45 KD ECP (**Figure 6B**). The EPS have been shown to consist of α-Rha, α-Glc, β-GlcNAc, and β-GalNAc (**Figure 6C**). Very recently, Vinogradov et al. (2016) showed that the structure of the EPS of *L. casei* BL23 consists of α-Rha, α-Glc, β-GlcNAc, and β-GalNAc, forming a branched heptasaccharide repeating unit (variant 1) with an additional partial substitution with α-Glc (variant 2) and a modified non-reducing octasaccharide end, corresponding to a terminal unit of the EPS (variant 3). We showed that the survival rate of zebrafish was significantly higher in fish treated with EPSP of BL23 at 10–20 μg/ml than in control fish after *A. veronii* infection (**Figure 5**, *P* < 0.001). In addition, the EPSP induced more expression of TLR1, TLR2, IL-10, and TNF-α and reduced the expression of IL-1β in ZFL cells (**Figure 7**, *P* < 0.05). These findings indicated that the EPSP enhanced zebrafish immune response against *A. veronii* might involve the TLR1/TLR2 signal pathway.

## Conclusion

Our results indicated that the *L. casei* BL23 showed high efficiency against *A. veronii* infection in zebrafish irrespective of cell viability. This protective effect of *L. casei* BL23 might involve membrane PRR signaling pathways induced by EPSP. Accordingly, *L. casei* BL23 may be suitable for disease control in aquaculture, especially for use in larval fish. However, gene knockout zebrafish lacking related factors are needed to further investigate the mechanisms underlying the action of EPSP.

## Author Contributions

All authors listed have made a substantial, direct and intellectual contribution to the work, and approved it for publication.

## Conflict of Interest Statement

The authors declare that the research was conducted in the absence of any commercial or financial relationships that could be construed as a potential conflict of interest.

## Footnotes

1 http://ccb.jhu.edu/software/FLASH/

## Supplementary Material

FIGURE S1 The anti-infective effect of potential protiotics against *Aeromonas veronii* in zebrafish larvae.

FIGURE S2 Gut microbiota of zebrafish from control or *Lactobacillus casei* BL23 treated fish at class **(A)**, order **(B)**, and family **(C)** level.

FIGURE S3 The mRNA levels of TLR3a **(A)**, TLR3b **(B)**, TLR4a **(C)**, TLR5a **(D)**, TLR5b **(E)**, MyD88 **(F)**, and NF-κB **(G)** in ZFL cells from control and Exopolysaccharide-protein complex (EPSP)-treated (10μg/mL) groups after 24 h treatment.

## References

[B1] Abbass A. Sharifuzzaman S. M. Austin B. (2010). Cellular components of probiotics control *Yersinia ruckeri* infection in rainbow trout, *Oncorhynchus mykiss* (Walbaum). *J. Fish Dis.* 33 31–37. 10.1111/j.1365-2761.2009.01086.x 19912460

[B2] Allen J. P. Neely M. N. (2010). Trolling for the ideal model host: zebrafish take the bait. *Future Microbiol.* 5 563–569. 10.2217/Fmb.10.24 20353298 PMC2885762

[B3] Beaz-Hidalgo R. Figueras M. J. (2013). *Aeromonas* spp. whole genomes and virulence factors implicated in fish disease. *J. Fish Dis.* 36 371–388. 10.1111/jfd.12025 23305319

[B4] Bleau C. Monges A. Rashidan K. Laverdure J. P. Lacroix M. Van Calsteren M. R. (2010). Intermediate chains of exopolysaccharides from *Lactobacillus rhamnosus* RW-9595M increase IL-10 production by macrophages. *J. Appl. Microbiol.* 108 666–675. 10.1111/j.1365-2672.2009.04450.x 19702865

[B5] Bondad-Reantaso M. G. Subasinghe R. P. Arthur J. R. Ogawa K. Chinabut S. Adlard R. (2005). Disease and health management in Asian aquaculture. *Vet. Parasitol.* 132 249–272. 10.1016/j.vetpar.2005.07.005 16099592

[B6] Bron P. A. van Baarlen P. Kleerebezem M. (2012). Emerging molecular insights into the interaction between probiotics and the host intestinal mucosa. *Nat. Rev. Microbiol.* 10 66–78. 10.1038/nrmicro2690 22101918

[B7] Caporaso J. G. Kuczynski J. Stombaugh J. Bittinger K. Bushman F. D. Costello E. K. (2010). QIIME allows analysis of high-throughput community sequencing data. *Nat. Methods* 7 335–336. 10.1038/nmeth.f.303 20383131 PMC3156573

[B8] Caporaso J. G. Lauber C. L. Walters W. A. Berg-Lyons D. Huntley J. Fierer N. (2012). Ultra-high-throughput microbial community analysis on the Illumina HiSeq and MiSeq platforms. *ISME J.* 6 1621–1624. 10.1038/ismej.2012.8 22402401 PMC3400413

[B9] Chabot S. Yu H. L. De Leseleuc L. Cloutier D. Van Calsteren M. R. Lessard M. (2001a). Exopolysaccharides from *Lactobacillus rhamnosus* RW-9595M stimulate TNF, IL-6 and IL-12 in human and mouse cultured immunocompetent cells, and IFN-gamma mouse splenocytes. *Lait* 81 683–697. 10.1051/lait:2001157

[B10] Chabot S. Yu H. L. Léséleuc L. D. Cloutier D. Calsteren M. R. V. Lessard M. (2001b). Exopolysaccharides from *Lactobacillus rhamnosus* RW-9595M stimulate TNF, IL-6 and IL-12 in human and mouse cultured immunocompetent cells, and IFN-$∖gamma$ in mouse splenocytes. *Dairy Sci. Technol.* 81 683–697. 10.1051/lait:2001157

[B11] Chen H. Liu S. Xu X. R. Liu S. S. Zhou G. J. Sun K. F. (2015). Antibiotics in typical marine aquaculture farms surrounding Hailing Island, South China: occurrence, bioaccumulation and human dietary exposure. *Mar. Pollut. Bull.* 90 181–187. 10.1016/j.marpolbul.2014.10.053 25467872

[B12] Cipriano R. C. Bullock G. L. Pyle S. W. (1984). *Aeromonas hydrophila and Motile Aeromonad Septicemias of Fish*. Washington, DC: United States Fish and Wildlife Service.

[B13] Edgar R. C. (2013). UPARSE: highly accurate OTU sequences from microbial amplicon reads. *Nat. Methods* 10 996–998. 10.1038/nmeth.2604 23955772

[B14] Emi Y. Masaki S. Tomoyuki S. (2008). Suppressive effect on activation of macrophages by *Lactobacillus casei* strain Shirota genes determining the synthesis of cell wall-associated polysaccharides. *Appl. Environ. Microbiol.* 74 4746–4755. 10.1128/AEM.00412-08 18552190 PMC2519339

[B15] FAO (2016). *The State of World Fisheries and Aquaculture 2016: Contributing to Food Security and Nutrition.* Rome: FAO.

[B16] Fečkaninová A. Koščová J. Mudroňová D. Popelka P. Toropilová J. (2017). The use of probiotic bacteria against *Aeromonas* infections in salmonid aquaculture. *Aquaculture* 469 1–8. 10.1016/j.aquaculture.2016.11.042

[B17] Fuente Mde L. Miranda C. D. Jopia P. Gonzalez-Rocha G. Guiliani N. Sossa K. (2015). Growth inhibition of bacterial fish pathogens and quorum-sensing blocking by bacteria recovered from Chilean salmonid farms. *J. Aquat. Anim. Health* 27 112–122. 10.1080/08997659.2014.1001534 26000731

[B18] Grangette C. Nutten S. Palumbo E. Morath S. Hermann C. Dewulf J. (2005). Enhanced antiinflammatory capacity of a *Lactobacillus plantarum* mutant synthesizing modified teichoic acids. *Proc. Natl. Acad. Sci. U.S.A.* 102 10321–10326. 10.1073/pnas.0504084102 15985548 PMC1177390

[B19] Hai N. V. (2015). The use of probiotics in aquaculture. *J. Appl. Microbiol.* 119 917–935. 10.1111/jam.12886 26119489

[B20] Huang Y. Zhang L. Tiu L. Wang H. H. (2015). Characterization of antibiotic resistance in commensal bacteria from an aquaculture ecosystem. *Front. Microbiol.* 6:914. 10.3389/fmicb.2015.00914 26441859 PMC4561822

[B21] Israngkura A. Haesae S. (2002). “A review of economic impacts of aquatic animal disease,” in *Primary Aquatic Animal Health Care in Rural, Small-scale Aquaculture Development, Technical Proceedings of the Asia Regional Scoping Workshop. FAO Fisheries Technical Paper 406* eds Arthur J. R. Phillips M. J. Subasinghe R. P. Reantaso M. B. McCrae I. H. (Rome: FAO) 55–61.

[B22] Kalliomaki M. Antoine J. M. Herz U. Rijkers G. T. Wells J. M. Mercenier A. (2010). Guidance for substantiating the evidence for beneficial effects of probiotics: prevention and management of allergic diseases by probiotics. *J. Nutr.* 140 713S–721S. 10.3945/jn.109.113761 20130079

[B23] Kanther M. Rawls J. F. (2010). Host-microbe interactions in the developing zebrafish. *Curr. Opin. Immunol.* 22 10–19. 10.1016/j.coi.2010.01.006 20153622 PMC3030977

[B24] Kim J. U. Kim Y. Han K. S. Whang K. Y. Kim J. N. Kim S. N. (2006). Function of cell-bound and released exopolysaccharides produced by *Lactobacillus rhamnosus* ATCC 9595. *J. Microbiol. Biotechnol.* 16 939–945.

[B25] Kim Y. Oh S. Kim S. H. (2009). Released exopolysaccharide (r-EPS) produced from probiotic bacteria reduce biofilm formation of enterohemorrhagic *Escherichia coli* O157:H7. *Biochem. Biophys. Res. Commun.* 379 324–329. 10.1016/j.bbrc.2008.12.053 19103165

[B26] Kitazawa H. Harata T. Uemura J. Saito T. Kaneko T. Itoh T. (1998). Phosphate group requirement for mitogenic activation of lymphocytes by an extracellular phosphopolysaccharide from *Lactobacillus delbrueckii* ssp. bulgaricus. *Int. J. Food Microbiol.* 40 169–175. 10.1016/S0168-1605(98)00030-0 9620124

[B27] Lafferty K. D. Harvell C. D. Conrad J. M. Friedman C. S. Kent M. L. Kuris A. M. (2015). Infectious diseases affect marine fisheries and aquaculture economics. *Ann. Rev. Mar. Sci.* 7 471–496. 10.1146/annurev-marine-010814-015646 25251276

[B28] Lebeer S. Vanderleyden J. De Keersmaecker S. C. (2010). Host interactions of probiotic bacterial surface molecules: comparison with commensals and pathogens. *Nat. Rev. Microbiol.* 8 171–184. 10.1038/nrmicro2297 20157338

[B29] Leung T. L. F. Bates A. E. Dulvy N. (2013). More rapid and severe disease outbreaks for aquaculture at the tropics: implications for food security. *J. Appl. Ecol.* 50 215–222. 10.1111/1365-2644.12017

[B30] Li-Li L. U. (2010). Probiotics in the prevention of antibiotics-associated diarrhea in children: a meta-analysis of randomized controlled trials. *J. Clin. Pediatr.* 42 367.e361–372.e361. 10.1016/j.jpeds.2006.04.053 16939749

[B31] Lin M. H. Yang Y. L. Chen Y. P. Hua K. F. Lu C. P. Sheu F. (2011). A novel exopolysaccharide from the biofilm of *Thermus aquaticus* YT-1 induces the immune response through Toll-like receptor 2. *J. Biol. Chem.* 286 17736–17745. 10.1074/jbc.M110.200113 21454596 PMC3093849

[B32] Mackenzie S. A. (2010). Peptidoglycan, not endotoxin, is the key mediator of cytokine gene expression induced in rainbow trout macrophages by crude LPS. *Mol. Immunol.* 47 1450–1457. 10.1016/j.molimm.2010.02.009 20304498

[B33] Magoc T. Salzberg S. L. (2011). FLASH: fast length adjustment of short reads to improve genome assemblies. *Bioinformatics* 27 2957–2963. 10.1093/bioinformatics/btr507 21903629 PMC3198573

[B34] Nagaoka M. Muto M. Nomoto K. Matuzaki T. Watanabe T. Yokokura T. (1990). Structure of polysaccharide-peptidoglycan complex from the cell wall of *Lactobacillus casei* YIT9018. *J. Biochem.* 108 568–571. 10.1093/oxfordjournals.jbchem.a123243 2292584

[B35] Nakajima H. Suzuki Y. Kaizu H. Hirota T. (2010). Cholesterol lowering activity of ropy fermented milk. *J. Food Sci.* 57 1327–1329. 10.1111/j.1365-2621.1992.tb06848.x

[B36] Nayak S. K. (2010). Probiotics and immunity: a fish perspective. *Fish Shellfish Immunol.* 29 2–14. 10.1016/j.fsi.2010.02.017 20219683

[B37] Newaj-Fyzul A. Al-Harbi A. H. Austin B. (2014). Review: developments in the use of probiotics for disease control in aquaculture. *Aquaculture* 431 1–11. 10.1016/j.aquaculture.2013.08.026

[B38] Pandiyan P. Balaraman D. Thirunavukkarasu R. George E. G. J. Subaramaniyan K. Manikkam S. (2013). Probiotics in aquaculture. *Drug Invent. Today* 5 55–59. 10.1016/j.dit.2013.03.003

[B39] Pereira A. M. P. T. Silva L. J. G. Meisel L. M. Pena A. (2015). Fluoroquinolones and tetracycline antibiotics in a Portuguese aquaculture system and aquatic surroundings: occurrence and environmental impact. *J. Toxicol. Environ. Health A* 78 959–975. 10.1080/15287394.2015.1036185 26262440

[B40] Pham L. N. Kanther M. Semova I. Rawls J. F. (2008). Methods for generating and colonizing gnotobiotic zebrafish. *Nat. Protoc.* 3 1862–1875. 10.1038/nprot.2008.186 19008873 PMC2596932

[B41] Phelps H. A. Neely M. N. (2005). Evolution of the zebrafish model: from development to immunity and infectious disease. *Zebrafish* 2 87–103. 10.1089/zeb.2005.2.87 18248169

[B42] Qin C. Xu L. Yang Y. He S. Dai Y. Zhao H. (2014). Comparison of fecundity and offspring immunity in zebrafish fed *Lactobacillus rhamnosus* CICC 6141 and *Lactobacillus casei* BL23. *Reproduction* 147 53–64. 10.1530/REP-13-0141 24129154

[B43] Reddy J. S. (2015). Probiotics in aquaculture: importance, influence and future perspectives. *Int. J. Bioassays* 4 3710–3718.

[B44] Romero J. Feijoó C. G. Navarrete P. (2012). “Antibiotics in aquaculture – use, abuse and alternatives,” in *Health Environment in Aquaculture* ed. Carvalho E. (Rijeka: InTech).

[B45] Ruasmadiedo P. Hugenholtz J. Zoon P. (2002). An overview of the functionality of exopolysaccharides produced by lactic acid bacteria. *Int. Dairy J.* 12 163–171. 10.1016/S0958-6946(01)00160-1 12647947

[B46] Sawada H. Furushiro M. Hirai K. Motoike M. Watanabe T. Yokokura T. (1990). Purification and characterization of an antihypertensive compound from *Lactobacillus casei*. *Agric. Biol. Chem.* 54 3211–3219. 10.1080/00021369.1990.10870492 1368639

[B47] Sazawal S. Hiremath G. Dhingra U. Malik P. Deb S. Black R. E. (2006). Efficacy of probiotics in prevention of acute diarrhoea: a meta-analysis of masked, randomised, placebo-controlled trials. *Lancet Infect. Dis.* 6 374–382. 10.1016/S1473-3099(06)70495-9 16728323

[B48] Selvaraju R. (2015). Beneficial and destructive effects of probiotics in aquaculture systems-A review. *Int. J. Fish. Aquat. Stud.* 2 153–159.

[B49] Sharifuzzaman S. M. Abbass A. Tinsley J. W. Austin B. (2011). Subcellular components of probiotics *Kocuria* SM1 and *Rhodococcus* SM2 induce protective immunity in rainbow trout (*Oncorhynchus mykiss*, Walbaum) against *Vibrio anguillarum*. *Fish Shellfish Immunol.* 30 347–353. 10.1016/j.fsi.2010.11.005 21078398

[B50] Stentiford G. D. Sritunyalucksana K. Flegel T. W. Williams B. A. Withyachumnarnkul B. Itsathitphaisarn O. (2017). New paradigms to help solve the global aquaculture disease crisis. *PLOS Pathog.* 13:e1006160. 10.1371/journal.ppat.1006160 28152043 PMC5289612

[B51] Subasinghe R. Soto D. Jia J. S. (2009). Global aquaculture and its role in sustainable development. *Rev. Aquac.* 1 2–9. 10.1111/j.1753-5131.2008.01002.x

[B52] Sullivan C. Kim C. H. (2008). Zebrafish as a model for infectious disease and immune function. *Fish Shellfish Immunol.* 25 341–350. 10.1016/j.fsi.2008.05.005 18640057

[B53] Tanaka T. Fujiwara S. Nishikori S. Fukui T. Takagi M. Imanaka T. (1999). A unique chitinase with dual active sites and triple substrate binding sites from the hyperthermophilic archaeon *Pyrococcus kodakaraensis* KOD1. *Appl. Environ. Microbiol.* 65 5338–5344. 10583986 10.1128/aem.65.12.5338-5344.1999 PMC91726

[B54] Trede N. S. Langenau D. M. Traver D. Look A. T. Zon I. L. (2004). The use of zebrafish to understand immunity. *Immunity* 20 367–379. 10.1016/S1074-7613(04)00084-6 15084267

[B55] Vinderola G. Perdigón G. Duarte J. Farnworth E. Matar C. (2006). Effects of the oral administration of the exopolysaccharide produced by *Lactobacillus kefiranofaciens* on the gut mucosal immunity. *Cytokine* 36 254–260. 10.1016/j.cyto.2007.01.003 17363262

[B56] Vinogradov E. Sadovskaya I. Grard T. Chapot-Chartier M. P. (2016). Structural studies of the rhamnose-rich cell wall polysaccharide of *Lactobacillus casei* BL23. *Carbohydr. Res.* 435 156–161. 10.1016/j.carres.2016.10.002 27756016

[B57] Wang Q. Garrity G. M. Tiedje J. M. Cole J. R. (2007). Naive Bayesian classifier for rapid assignment of rRNA sequences into the new bacterial taxonomy. *Appl. Environ. Microbiol.* 73 5261–5267. 10.1128/Aem.00062-07 17586664 PMC1950982

[B58] Welman A. D. Maddox I. S. (2003). Exopolysaccharides from lactic acid bacteria: perspectives and challenges. *Trends Biotechnol.* 21 269–274. 10.1016/S0167-7799(03)00107-0 12788547

[B59] World Bank (2014). *Reducing Disease Risk in Aquaculture.* Washington, DC: World Bank 1–119.

[B60] Wu M. H. Pan T. M. Wu Y. J. Chang S. J. Chang M. S. Hu C. Y. (2010). Exopolysaccharide activities from probiotic bifidobacterium: immunomodulatory effects (on J774A.1 macrophages) and antimicrobial properties. *Int. J. Food Microbiol.* 144 104–110. 10.1016/j.ijfoodmicro.2010.09.003 20884069

[B61] Zhang Z. Zhou Z. G. Li Y. Zhou L. K. Ding Q. W. Xu L. (2016). Isolated exopolysaccharides from *Lactobacillus rhamnosus* GG alleviated adipogenesis mediated by TLR2 in mice. *Sci. Rep.* 6:36083. 10.1038/srep36083 27786292 PMC5081535

