# Supplementary figures and images for: EPSP of L. casei BL23 Protected against the Infection Caused by Aeromonas veronii via Enhancement of Immune Response in Zebrafish
Source: Front Microbiol. 2017 Dec 8;8:2406. doi: 10.3389/fmicb.2017.02406 (PMC5770644; doi:10.3389/fmicb.2017.02406)

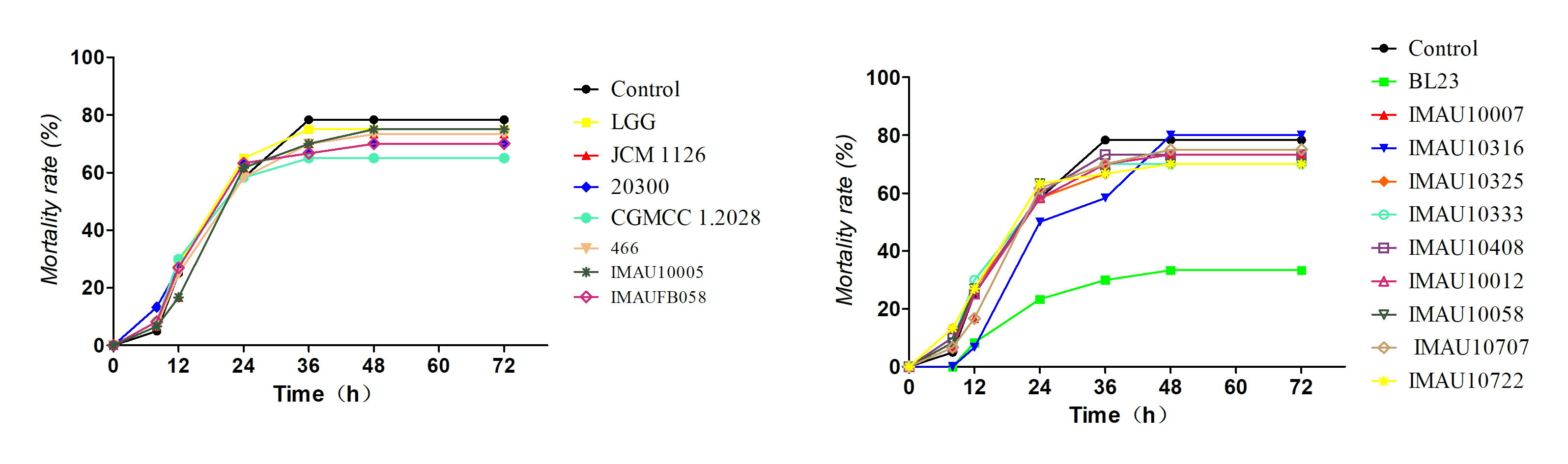

Supplement: FIGURE S1 — The anti-infective effect of potential protiotics against Aeromonas veronii in zebrafish larvae. [file Image_1.tif]

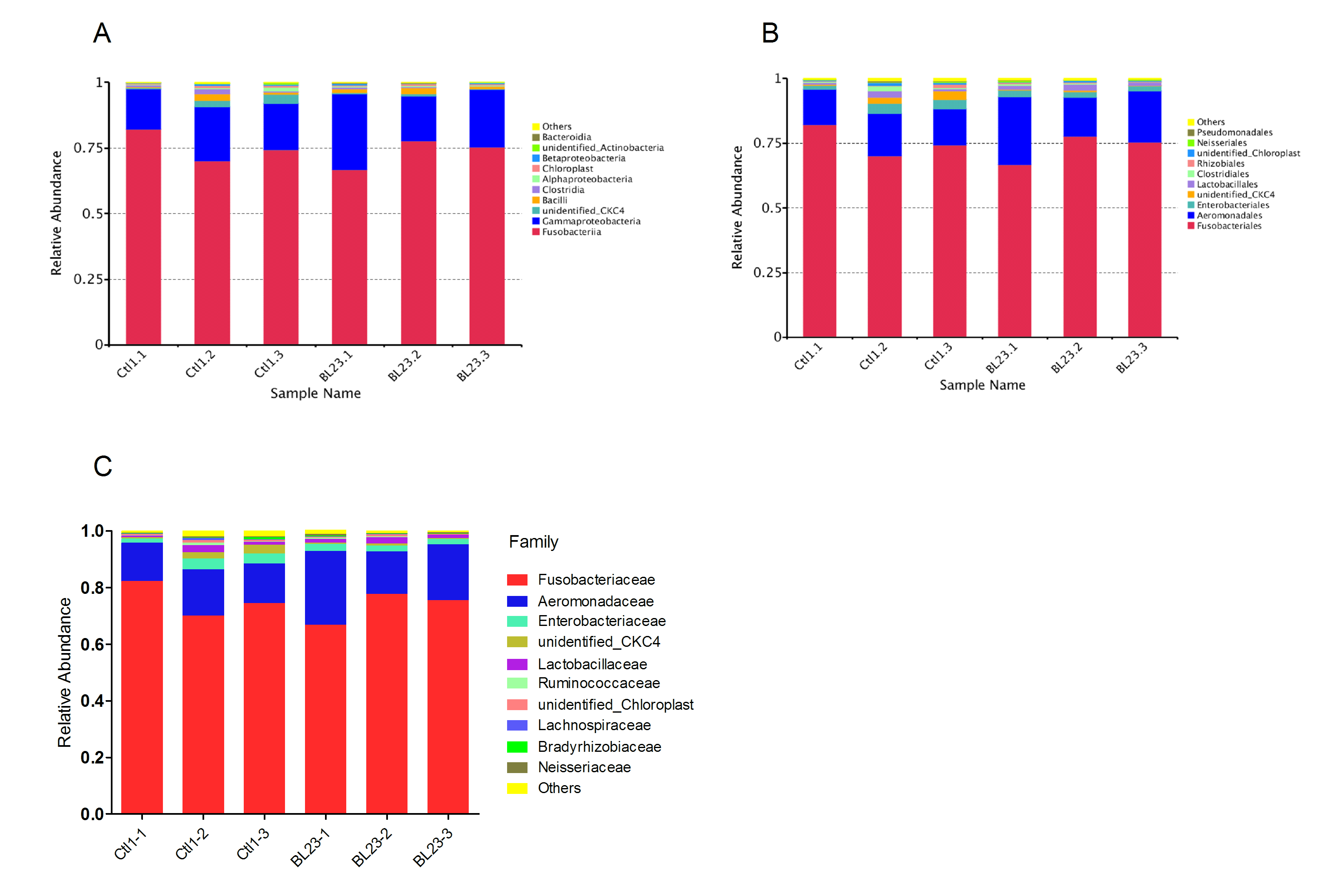

Supplement: FIGURE S2 — Gut microbiota of zebrafish from control or Lactobacillus casei BL23 treated fish at class (A), order (B), and family (C) level. [file Image_2.TIF]

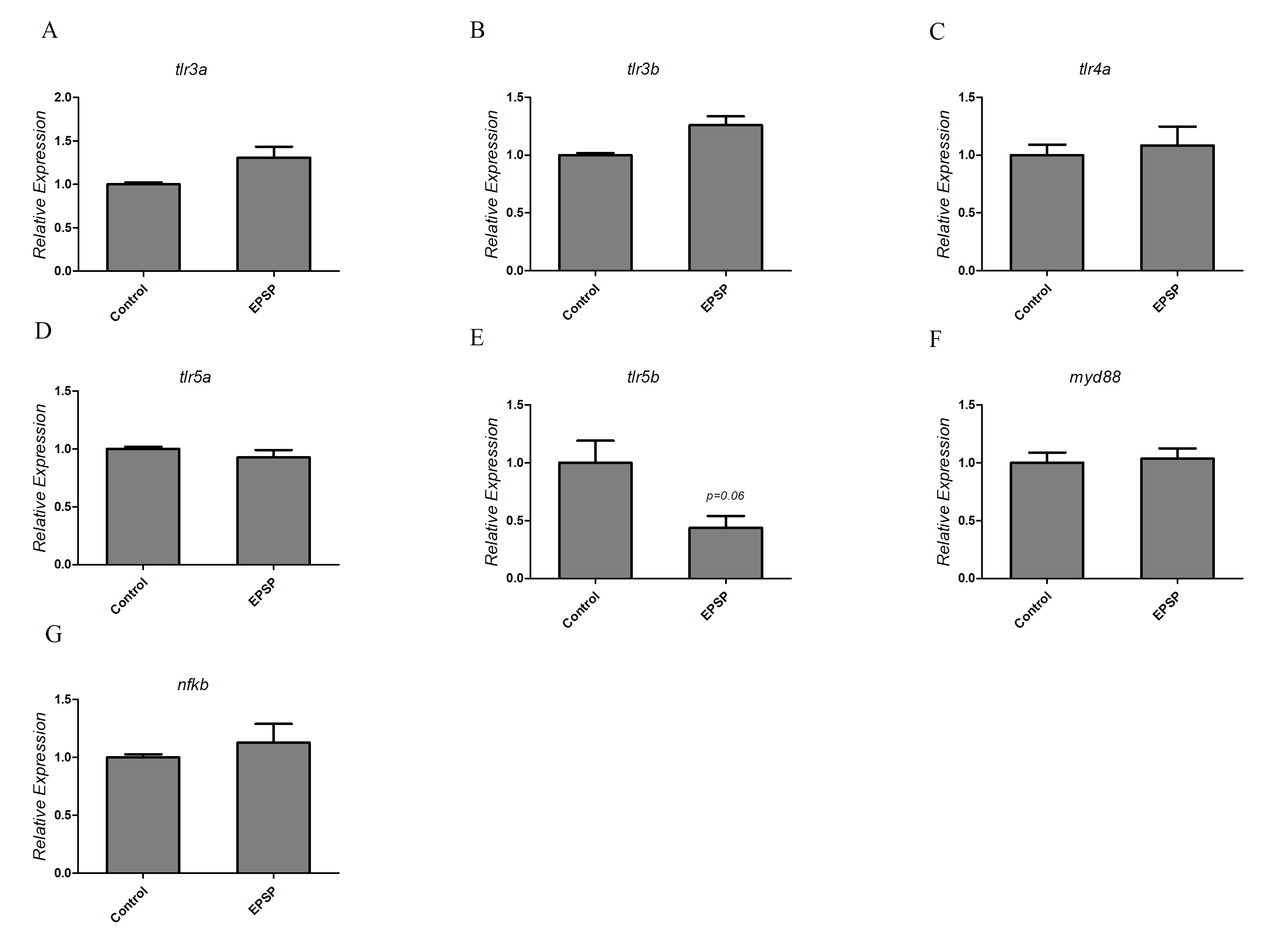

Supplement: FIGURE S3 — The mRNA levels of TLR3a (A), TLR3b (B), TLR4a (C), TLR5a (D), TLR5b (E), MyD88 (F), and NF-κB (G) in ZFL cells from control and Exopolysaccharide-protein complex (EPSP)-treated (10μg/mL) groups after 24 h treatment. [file Image_3.tif]
